# Comparison of Two Creatinine-Based Equations for Predicting Decline in Renal Function in Type 2 Diabetic Patients with Nephropathy in a Korean Population

**DOI:** 10.1155/2013/848963

**Published:** 2013-12-24

**Authors:** Eun Young Lee, Young-Mi Lee, Kyu Hun Choi, Hyun Chul Lee, Byung-Wan Lee, Beom Seok Kim

**Affiliations:** ^1^Department of Internal Medicine, Yonsei University College of Medicine, 50 Yonsei-ro, Seodaemun-gu, Seoul 120-752, Republic of Korea; ^2^Department of Internal Medicine, Dongtan Jeil Women's Hospital, 42-1 Seokwoo-dong, Hwasung, Gyeonggi-do 445-170, Republic of Korea; ^3^Division of Nephrology, Department of Internal Medicine, Yonsei University College of Medicine, 50 Yonsei-ro, Seodaemun-gu, Seoul 120-752, Republic of Korea; ^4^Division of Endocrinology and Metabolism, Department of Internal Medicine, Yonsei University College of Medicine, 50 Yonsei-ro, Seodaemun-gu, Seoul 120-752, Republic of Korea

## Abstract

*Aim.* To compare two creatinine-based estimated glomerular filtration rate (eGFR) equations, the chronic kidney disease epidemiology collaboration (CKD-EPI) and the modification of diet in renal disease (MDRD), for predicting the risk of CKD progression in type 2 diabetic patients with nephropathy. *Methods.* A total of 707 type 2 diabetic patients with 24 hr urinary albumin excretion of more than 30 mg/day were retrospectively recruited and traced until doubling of baseline serum creatinine (SCr) levels was noted. *Results.* During the follow-up period (median, 2.4 years), the CKD-EPI equation reclassified 10.9% of all MDRD-estimated subjects: 9.1% to an earlier stage of CKD and 1.8% to a later stage of CKD. Overall, the prevalence of CKD (eGFR < 60 mL/min/1.73 m^2^) was lowered from 54% to 51.6% by applying the CKD-EPI equation. On Cox-regression analysis, both equations exhibited significant associations with an increased risk for doubling of SCr. However, only the CKD-EPI equation maintained a significant hazard ratio for doubling of SCr in earlier-stage CKD (eGFR ≥ 45 mL/min/1.73 m^2^), when compared to stage 1 CKD (eGFR ≥ 90 mL/min/1.73 m^2^). *Conclusion.* In regard to CKD progression, these results suggest that the CKD-EPI equation might more accurately stratify earlier-stage CKD among type 2 diabetic patients with nephropathy than the MDRD study equation.

## 1. Introduction

An increasing prevalence of chronic kidney disease (CKD) is garnering greater concern worldwide [1]. Previous studies have attributed a growing trend in CKD to a rapid aging of the general population and expansion of the diabetes epidemic [1–3]. From 1991 to 2001, the incidence of diabetic nephropathy doubled among patients with newly diagnosed end stage renal disease (ESRD) [4]. Recently, an outstanding cross-sectional study including 32,208 patients with type 2 diabetes (T2D) from 33 countries revealed that the overall prevalence of micro- and macroalbuminuria was about 39% and 10%, respectively [5]. Making matters worse, the prevalence of ESRD caused by diabetes is estimated to increase to 70% by the year 2015 [4]. T2D is well known as a leading cause of cardiovascular disease (CVD) and ESRD [6]. It is also well established that CKD has been shown to be strongly related to increased risks of CVD-related hospitalization and mortality, as well as ESRD, even after adjusting cardiovascular risk factors [1, 7, 8]. Therefore, early identification of patients with CKD may hold additional clinical implications other than just the detection of impending progression to ESRD, especially in patients with T2D [9, 10].

Diagnosis, classification, and management of CKD are mainly dependent on overall kidney function assessed by glomerular filtration rate (GFR). To aid in the above, several creatinine-based formulas have been developed for estimating GFR. Most widely used in clinical practice, the modification of diet in renal disease (MDRD) study equation for estimating GFR was developed accounting for serum creatinine concentration, age, sex, and race [1]. Via subsequent studies, the prognostic implications of estimated GFR (eGFR) based on the MDRD study equation (eGFR MDRD) were revealed [1, 11, 12]. In accordance with these reports, eGFR MDRD has been widely known to predict the risk of ESRD in CKD patients, as well as graft failure after kidney transplant [13, 14]. Moreover, decline in eGFR MDRD has also been reported to be predictive of clinical outcomes, such as CVD events and death, particularly in patients with a CKD (GFR < 60 mL/min/1.73 m^2^) [1, 15, 16]. Although the MDRD study equation has generally been used for estimating GFR and evaluating CKD, imprecision and underestimation of GFR have been reported as major limitations, especially in those with early stage of CKD (GFR ≥ 60 mL/min/1.73 m^2^) [1, 11, 12]. In addition, in diabetic patients with microalbuminuria or overt diabetic nephropathy, it was reported that the MDRD Study equation significantly underestimated GFR [17]. Recently, the chronic kidney disease epidemiology collaboration (CKD-EPI) equation was developed utilizing a large database that pooled data from 10 studies and has been subsequently validated in 16 additional studies [18]. Based on the same four variables of the MDRD study equation, age, sex, race, and serum creatinine concentration, the CKD-EPI equation has proven to be more accurate than the MDRD Study equation in estimating GFR, especially in patients with early stage of CKD. However, there have been few studies to compare the CKD-EPI and MDRD equations with respect to the risks of clinical outcomes such as loss of kidney function or progression to ESRD in patients with T2D. Therefore, we attempted to investigate whether the CKD-EPI equation was superior to the MDRD equation in predicting decline in renal function in Korean type 2 diabetic patients with nephropathy.

## 2. Materials and Methods

### 2.1. Patients and Study Design

In this retrospective cohort study, we extracted data from an electronic medical record (EMR) database of type 2 diabetic subjects with nephropathy in whom two or more serum creatinine measurements were made between July 2000 and September 2012 at Severance Hospital in Seoul, Korea. Patients with type 2 diabetes were identified by searching the EMR database for the code ICD-10. Indicative of diabetic nephropathy, we included diabetic patients with 24 hr urinary albumin excretion ≥ 30mg/day on at least one measurement. Baseline data were defined as data measured at the point in time at which 24 hr urinary albumin excretion level exceeded 30 mg/day for the first time. Patients were excluded if they had undergone renal replacement therapy at baseline or if they were younger than 18 years. After the baseline data extraction, patients were retrospectively followed up to two set endpoints: until May 2013 (time endpoint) or until an event of decline in renal function or death (clinical outcome endpoint). For subjects who were lost to follow-up, we included data obtained up to their final visit.

Primary outcome was evaluated according to decline in renal function and defined as doubling of baseline serum creatinine level. Doubling of baseline serum creatinine level was defined as a twofold increase in serum creatinine level for at least two consecutive measurements. This study was approved by the Institutional Review Board of Severance Hospital.

### 2.2. Clinical and Laboratory Measurements

Demographic and clinical findings were reviewed retrospectively for age, gender, duration of diabetes, and medications. Body mass index (BMI, kg/m^2^) was calculated by dividing weight (kg) by height (m) squared. Urinary albumin excretion amounts were measured with an automatic analyzer, Hitachi 7180 (Hitachi Instruments Service, Tokyo, Japan), in a 24 hr urine sample. Plasma glucose level was determined by the glucose oxidase method. HbA1c was measured by high-performance liquid chromatography using the Variant II Turbo Hemoglobin Testing System (Bio-Rad Laboratories, Hercules, CA). Plasma total cholesterol, high-density lipoprotein (HDL) cholesterol, triglycerides (TG), and creatinine measurements were performed using an autoanalyzer (Hitachi 7600: Hitachi Instruments Service, Tokyo, Japan). Low-density lipoprotein (LDL) cholesterol was calculated using the Friedewald formula.

### 2.3. Estimation of GFR and Classification of CKD

The estimation of GFR was calculated using the four-variable MDRD study equation and the CKD-EPI equation [18, 19]:

(1) eGFR(MDRD)=186.3×(creatinine)−1.154×Age−0.203×0.742 (if  female),eGFR(CKD-EPI)=141×min⁡(creatininek,1)α×max⁡⁡(creatininek,1)−1.209×0.993Age×1.018 (if  female).

In the CKD-EPI equation for estimating GFR, *k* equals 0.7 for females and 0.9 for males; *α* equals −0.329 for females and −0.411 for males; min refers to the minimum value for creatinine/*k* or 1; and max means the maximum for creatinine/*k* or 1. For both equations, eGFR was calculated as mL/min/1.73 m^2^, weight in kg, serum creatinine in mg/dL, and age in years. CKD stage was classified into five subgroups according to the NKF-KDOQI criteria for CKD: stage 1, eGFR ≥ 90 mL/min/1.73 m^2^; stage 2, eGFR of 60–89 mL/min/1.73 m^2^; stage 3, eGFR of 30–59 mL/min/1.73 m^2^; stage 4, eGFR of 15–29 mL/min/1.73 m^2^; and stage 5, eGFR < 15 mL/min/1.73 m^2^or dialysis. Stage 3 CKD was further divided into two subgroups: stage 3a, eGFR 45–59 mL/min/1.73 m^2^, and stage 3b, eGFR 30–44 mL/min/1.73 m^2^ [12, 20].

### 2.4. Statistical Analysis

Data are presented as the means ± standard deviation. CKD was defined as an eGFR < 60 mL/min/1.73 m^2^ for both equations for eGFR calculation [12]. Analysis of the associations between eGFR calculated by each equation and the risk of clinical outcomes was performed with Cox regression analysis after adjusting for potential confounding factors, including age, sex, diabetes duration, and HbA1c. All statistical analyses were performed with SAS version 9.2 (SAS Institute, Cary, NC, USA), and *P* values <0.05 were considered statistically significant.

## 3. Results

The baseline characteristics of all 707 subjects are shown in Table 1. Mean age, HbA1c, and duration of diabetes were 61.9 ± 12.2 years, 8.2 ± 4.3 %, and 12.7 ± 8.9 years, respectively. The mean 24 hr urinary albumin excretion amount was 1094.58 ± 1867.38 mg/day, and 47.5% of the patients exhibited macroalbuminuria. The prevalence of CKD, defined as an eGFR of less than 60 mL/min/1.73 m^2^, was 54% (*n* = 382) for the MDRD study equation and 51.6%, (*n* = 365) for the CKD-PEI equation. Oral antidiabetic drugs and insulin were used in 69.9% and 26.0% of all patients, respectively. In this study, 68.2% and 37.9% of the subjects had also taken medication for hypertension and dyslipidemia, respectively.

**Figure 1:**
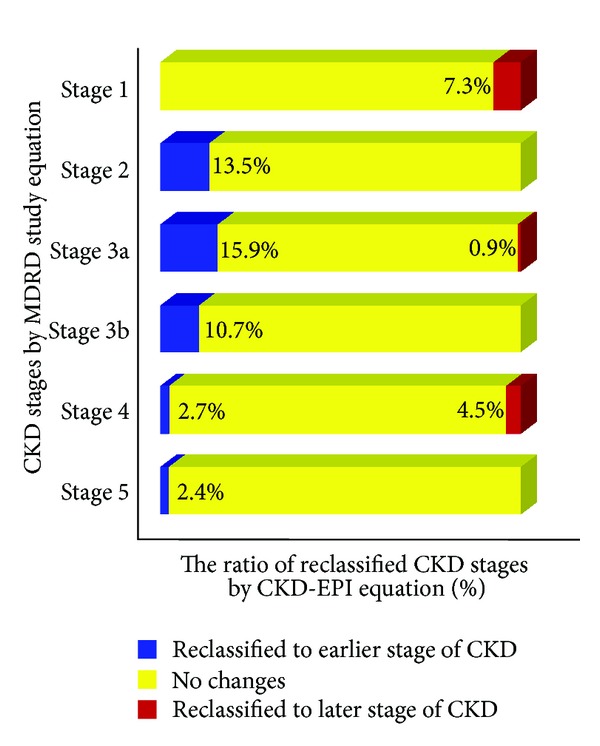
Ratio (%) of chronic kidney disease (CKD) stages for the modification of diet in renal disease (MDRD) study equation reclassified by the chronic kidney disease epidemiology collaboration (CKD-EPI) equation. Blue bars indicate reclassification to earlier stage of CKD; yellow bars, no reclassification; red bars, reclassification to later stage of CKD.

The most common CKD stage was stage 2 for both the MDRD study equation and the CKD-EPI equation (Table 1). Comparing CKD stage for each equation, 10.9% of MDRD-estimated patients were reclassified by adopting the CKD-EPI equation. Most reclassifications of CKD stage were observed in patients with stage 3a (eGFR MDRD of 45–59 mL/min/1.73 m^2^) (Figure 1). Among these patients (*n* = 107), 15.9% (*n* = 17) were reclassified to a lower stage of CKD and 0.9% were reclassified to a higher stage of CKD. Of the 229 patients with CKD stage 2 by eGFR MDRD, 13.5% (*n* = 31) were downwardly reclassified to CKD stage 1 by eGFR CKD-EPI, lowering the prevalence of CKD stage 2 from 32.4% to 31.4%. In CKD stage 3b patients with an eGFR MDRD of 30–44 mL/min/1.73 m^2^, upward reclassification to CKD stage 3a by eGFR CKD-EPI occurred in 10.7% (*n* = 13) of 122 patients. In contrast, 7.3% (*n* = 7) of 96 patients with CKD stage 1 by eGFR MDRD were upwardly reclassified to CKD stage 2 by eGFR CKD-EPI. Overall, the prevalence of CKD (defined as an eGFR of less than 60 mL/min/1.73 m^2^) decreased from 54% to 51.6% by applying the CKD-EPI equation. Additionally, reclassification to an earlier stage of CKD by applying the CKD-EPI equation was likely to occur in younger subjects (median age, 55.5 versus 64.2 years, *P* < 0.001); patients reclassified to a later stage of CKD by the CKD-EPI equation were older than those who were not reclassified (median age, 76.8 versus 64.2 years, *P* < 0.001).

**Figure 2:**
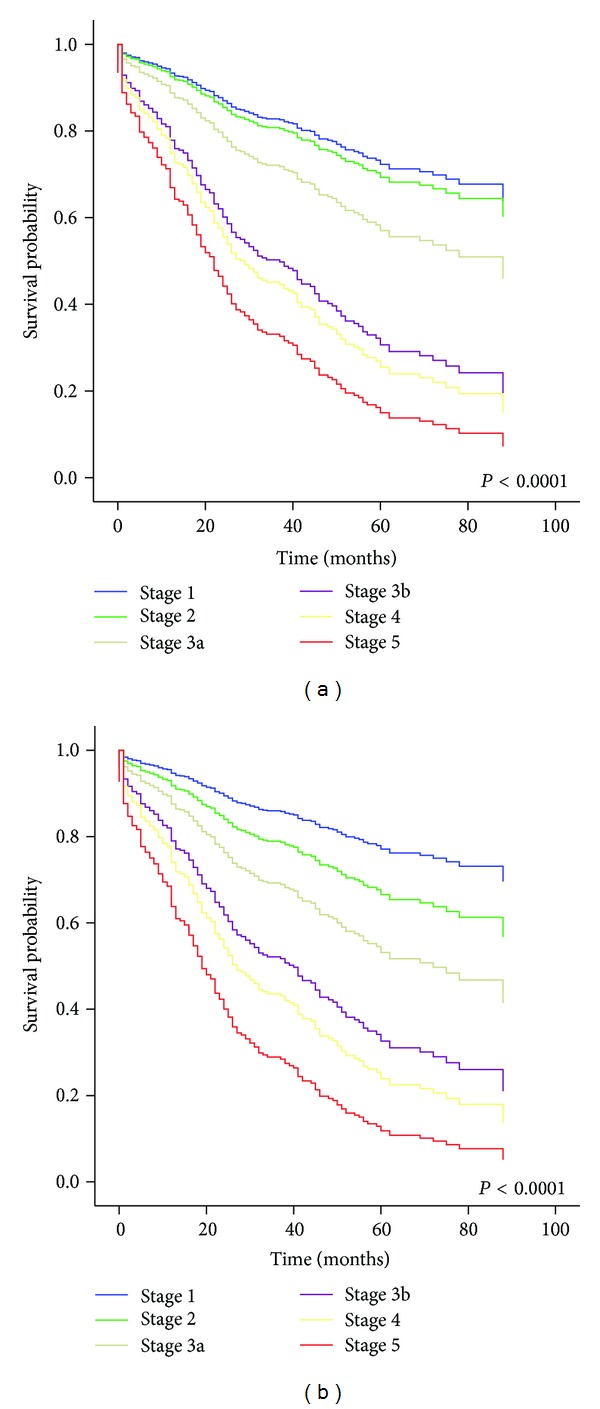
Cox-regression survival curve for doubling of serum creatinine level according to CKD stage for each eGFR equation: MDRD (a) and CKD-EPI (b). Age and sex are adjusted.

During a median follow-up of 2.4 years, doubling of serum creatinine level, development of ESRD, incidence of acute myocardial infarction (AMI), and stroke, as well as death from any cause, occurred in 27.9 % (*n* = 197), 13.4% (*n* = 95), 6.5% (*n* = 46), 6.5% (*n* = 46), and 10.7% (*n* = 76) of the participants, respectively. As shown in Figure 2, advance of CKD stage for both equations was associated with an increased risk for doubling of serum creatinine level in a stage-dependent manner. In Cox regression analyses (Table 2), CKD stage classified by each equation was associated with an increased risk for doubling of baseline serum creatinine levels. Comparing all CKD stages to stage 1 CKD estimated by both equations, we assessed Cox proportional hazard ratios (HRs) for doubling of serum creatinine level. For the MDRD study equation, the Cox proportional HRs for doubling of serum creatinine level were 1.54 (95% CI, 0.71–3.31; *P *= 0.27) for stage 2 and 1.79 (95% CI, 0.79–4.07; *P *= 0.17) for stage 3a. In contrast, for the CKD-EPI equation, the Cox proportional HRs for doubling of serum creatinine level were 1.90 (95% CI, 0.97–3.73; *P *= 0.063) for stage 2 and 2.18 (95% CI, 1.04–4.55; *P *= 0.038) for stage 3a. In the advanced stages of CKD (eGFR < 45 mL/min/1.73 m^2^), both equations showed significant HRs for doubling of serum creatinine level. In model 1, the association of decreased eGFR estimated by each equation with the risk of baseline creatinine level doubling remained statistically significant after adjusting for age and sex. Additionally adjusting for duration of diabetes in model 2, duration of diabetes and HbA1c in model 3, and further adjusting for medication for hypertension in model 4, in both Cox regression models, eGFR CKD-EPI showed greater HRs for doubling of serum creatinine level than eGFR MDRD did.

## 4. Discussion

Considering the burden of CKD on public health worldwide, accurate estimation of renal function is of paramount importance in managing subjects with renal insufficiency, as well as in improving morbidity and mortality [18, 21]. Current clinical guidelines for CKD recommend reporting serum creatinine-based eGFR using the MDRD study formula, which includes data for age, sex, race, and serum creatinine concentration [12, 22]. Based on data from patients with CKD, the MDRD study equation is limited by imprecision and underestimation of GFR in patients of early stage of CKD (GFR ≥ 60 mL/min/1.73 m^2^) [23, 24]. Because of these challenges, application of the MDRD study equation is considered less useful to classify patients of CKD stages 1 and 2, to verify hyperfiltration, and to track GFR changes in the higher range [1]. Furthermore, it is reported that eGFR assessed by the MDRD study equation overdiagnosed CKD, especially in younger white women [24, 25]. A recent meta-analysis, based on various populations, revealed that not only the classification of CKD but also the risk for mortality and ESRD were more accurately predicted by the CKD-EPI equation than the MDRD study equation [26]. These unmet needs drove the advent of a new equation proposed by the CKD-EPI. Growing evidence has demonstrated that the CKD-EPI equation might be more accurate than the MDRD study equation [18, 27–29]. However, the clinical implications of eGFR assessed by the CKD-EPI equation compared to that by the MDRD study equation have not yet been well elucidated in Korean subjects with type 2 diabetes.

The present study demonstrated the superiority of the CKD-EPI equation over the MDRD study equation in identifying Korean type 2 diabetic subjects with nephropathy who were expected to show deteriorations in renal function. The present study had two main findings: first, compared with the results from eGFR MDRD, 9.1% of the type 2 diabetic subjects with nephropathy were reclassified to an earlier stage of CKD after estimation of GFR by the CKD-EPI equation. This resulted in a decrease in the prevalence of CKD stages 2, 3, and 4 from 32.4% to 31.4%, 32.4% to 30.4%, and 15.7% to 14.7%, respectively. With respect to discrepancies in CKD stage between eGFR CKD-EPI and eGFR MDRD, most studies have reported similar trends in decreased prevalence of CKD (eGFR < 60 mL/min/1.73 m^2^) estimated by the CKD-EPI equation in comparison to the MDRD study equation [18, 28]. Regarding precision and accuracy, one previous study demonstrated that eGFR MDRD was imprecise in patients with an eGFR ≥ 60 mL/min/1.73 m^2^, while eGFR CKD-EPI showed less bias, improved precision, and greater accuracy than eGFR MDRD [30]. In accordance with this finding, introduction of the CKD-EPI equation in the National Health and Nutrition Examination Survey (NHANES) led to a decrease in the estimated prevalence of CKD from 13.1% to 11.5% [18]. Furthermore, in a recently conducted cohort study for subjects with T2D, the prevalence of CKD (eGFR < 60 mL/min/1.73 m^2^) was 22.0% for eGFR MDRD and 20.2% for eGFR CKD-EPI [31]. Because we targeted type 2 diabetic patients with nephropathy in the present study, the prevalence rate of CKD was nearly twice as high as that of the previous study. Nonetheless, similar to the previous study, we observed a decrease in the prevalence of CKD (eGFR < 60 mL/min/1.73 m^2^) from 54.0% to 51.6% when estimated by the CKD-EPI equation. Accordingly, we deduced that the CKD-EPI equation might allow for better risk assessment and more effective use of health care resources allocated to managing CKD-related outcomes, owing to a lower and more accurately assessed CKD prevalence [32]. Second, only the CKD-EPI equation, not the MDRD study equation, was able to predict the progression of renal insufficiency in type 2 diabetic subjects who already had albuminuria and earlier stage of CKD (GFR ≥ 45 mL/min/1.73 m^2^). As the prevalence of earlier-stage CKD (10.8%) is more than 100 times greater than the prevalence of renal failure (0.1%), more accurate detection of CKD at earlier stages could help not only in clinical decision making but also in the allocation of public health care resources, as stated above [9]. In this regard, improvement in the early prediction of decline in renal function before it develops to ESRD might have significant clinical implications.

Recently, several studies in general population cohorts have demonstrated that reclassification of eGFR by the CKD-EPI equation facilitates more accurate prediction of clinical outcomes than assessment of eGFR by the MDRD study equation, in particular by shifting lower risk participants to an earlier stage of CKD [27–29]. As a practical point, the way to demonstrate the accuracy of a particular method for estimating GFR may be to evaluate its ability to predict adverse clinical outcomes [27, 32]. In other words, improvement in the ability of an eGFR equation to predict adverse outcomes may reflect more accurate estimation of GFR by said equation. In this regard, the CKD-EPI equation might be the most accurate method for estimating GFR in various populations [1, 18, 30]. Despite increasing evidence of the merits of the CKD-EPI equation, only one study to date has been performed comparing the MDRD study equation with the CKD-EPI equation for predicting adverse clinical outcomes in type 2 diabetic patients, in which the CKD-EPI equation reportedly predicted mortality more accurately than the MDRD study equation did [31]. However, in the present study, we failed to observe a significant difference in predicting all-cause mortality, ESRD, AMI, or stroke (data not shown). This might be due to the relatively small sample size and short follow-up times (median follow-up: 2.4 years). Overall, our results suggest that improved estimation of GFR by the CKD-EPI equation, compared to the MDRD study equation, allowed for better risk categorization for decline in renal function in T2D patients in Korea.

In addition to the retrospective nature of our study, there are a few important limitations that warrant consideration. First, we did not evaluate the accuracy of the two eGFR equations for estimating GFR in type 2 diabetic patients with nephropathy in comparison with directly measured GFR (e.g., GFR measurement by using inulin or isotope). Second, although the CKD-EPI equation holds greater clinical implications than the MDRD study equation in patients of an earlier stage of CKD, it still involves the inherent limitations of serum creatinine, which is dependent on muscle mass, generation, and tubular secretion [11]. Thirdly, we followed up the subjects for a relatively short term (median, 2.4 years) and had no information about potential confounding factors (e.g., smoking). Therefore, some important clinical outcomes such as ESRD or mortality could not be appropriately evaluated. Lastly, this study comprised only Korean patients with T2D, preventing our results from being generalized to other ethnic populations.

Regarding management of subjects with T2D, long-term medical complications such as CVD and ESRD should be taken into account, especially in those with diabetic nephropathy who are more prone to deteriorations in renal function and are at higher risk for comorbidities, such as CVD and mortality. In this regard, accurate prediction for possible progression to renal failure might be one of the most important clinical endpoints in evaluating diabetic patients who show the potential for unwanted clinical outcomes. In accordance with previous reports and our results, the CKD-EPI equation could be considered an optimal equation in evaluating persons with normal renal function or earlier stage of CKD, a clinical scenario similar to early stage diabetic nephropathy [18, 27–29]. Taken together, our findings, despite their limitations, may hold several clinical implications that warrant further investigation.

## 5. Conclusions

In conclusion, we suggest that the CKD-EPI equation is superior to the MDRD study equation in identifying type 2 diabetic subjects with nephropathy prone to decline in renal function. However, further studies are needed to verify the accuracy and precision of the CKD-EPI equation compared to the MDRD study equation in estimating GFR in more diverse populations such as elderly patients, different ethnic groups, and patients with T2D [18, 33].

## Figures and Tables

**Table 1:** Demographic and baseline characteristics of the participants (*n* = 707).

Variables	All
Male/female	416/291
Age (years)	61.9 ± 12.2
Duration of diabetes (years)	12.7 ± 8.9
Duration of follow-up (months)	35.9 ± 49.6
HbA1c (%)	8.2 ± 4.3
Cr (mg/dL)	1.59 ± 1.24
eGFR CKD-EPI(mL/min/1.73 m^2^)	59.08 ± 30.27
Stages, *n* (%)	
Stage 1: ≥90	120 (17.0)
Stage 2: 60–89	222 (31.4)
Stage 3a: 45–59	102 (14.4)
Stage 3b: 30–44	113 (16.0)
Stage 4: 15–29	104 (14.7)
Stage 5: <15	46 (6.5)
eGFR MDRD (mL/min/1.73 m^2^)	58.01 ± 31.77
Stages, *n* (%)	
Stage 1: ≥90	96 (13.6)
Stage 2: 60–89	229 (32.4)
Stage 3a: 45–59	107 (15.1)
Stage 3b: 30–44	122 (17.3)
Stage 4: 15–29	111 (15.7)
Stage 5: <15	42 (5.9)
24 hr urinary albumin excretion (mg/day)	1094.58 ± 1867.38
Total cholesterol (mg/dL)	174.1 ± 53.9
Triglycerides (mg/dL)	169.6 ± 140.4
HDL cholesterol (mg/dL)	43.5 ± 15.1
LDL cholesterol (mg/dL)	99.3 ± 42.2
Oral hypoglycemic agents (%)	495 (69.9)
Insulin therapy (%)	184 (26.0)
Antihypertensive agents (%)	483 (68.2)
Lipid-lowering agents (%)	268 (37.9)

Data are shown as means ± SD or number of the case (%).

**Table 2:** Crude and adjusted Cox proportional hazard ratios for doubling of baseline serum creatinine level in 707 type 2 diabetic patients with nephropathy stratified by CKD stage according to each equation.

	HR	95% CI	*P*		HR	95% CI	*P*
CKD_MDRD_			<0.0001	CKD_CKD-EPI_			<0.0001
Stage 1: ≥90	1	Reference		Stage 1: ≥90	1	Reference	
Stage 2: 60–89	1.54	0.72–3.32	0.2679	Stage 2: 60–89	1.90	0.97–3.73	0.0630
Stage 3a: 45–59	1.79	0.79–4.07	0.1658	Stage 3a: 45–59	2.18	1.04–4.55	0.0383
Stage 3b: 30–44	4.11	1.92–8.82	0.0003	Stage 3b: 30–44	4.31	2.19–8.48	<0.0001
Stage 4: 15–29	4.40	2.04–9.49	0.0002	Stage 4: 15–29	5.08	2.56–10.08	<0.0001
Stage 5: <15	6.64	2.66–16.61	<0.0001	Stage 5: <15	8.09	3.65–17.95	<0.0001

Adjusted model 1			<0.0001	Adjusted model 1			<0.0001
Stage 1: ≥90	1	Reference		Stage 1: ≥90	1	Reference	
Stage 2: 60–89	1.13	0.57–2.21	0.7311	Stage 2: 60–89	1.56	0.84–2.89	0.1564
Stage 3a: 45–59	1.73	0.85–3.51	0.1324	Stage 3a: 45–59	2.42	1.26–4.67	0.0083
Stage 3b: 30–44	3.63	1.83–7.18	0.0002	Stage 3b: 30–44	4.29	2.29–8.02	<0.0001
Stage 4: 15–29	4.20	2.10–8.37	<0.0001	Stage 4: 15–29	5.47	2.89–10.35	<0.0001
Stage 5: <15	5.83	2.62–12.98	<0.0001	Stage 5: <15	8.17	3.98–16.78	<0.0001

Adjusted model 2			<0.0001	Adjusted model 2			<0.0001
Stage 1: ≥90	1	Reference		Stage 1: ≥90	1	Reference	
Stage 2: 60–89	1.12	0.55–2.28	0.7493	Stage 2: 60–89	1.70	0.88–3.28	0.1139
Stage 3a: 45–59	1.63	0.77–3.43	0.2013	Stage 3a: 45–59	2.32	1.14–4.70	0.0199
Stage 3b: 30–44	3.40	1.64–7.07	0.0010	Stage 3b: 30–44	4.39	2.22–8.67	<0.0001
Stage 4: 15–29	3.60	1.73–7.51	0.0006	Stage 4: 15–29	5.08	2.55–10.13	<0.0001
Stage 5: <15	5.34	2.27–12.55	0.0001	Stage 5: <15	7.79	3.57–17.02	<0.0001

Adjusted model 3			<0.0001	Adjusted model 3			<0.0001
Stage 1: ≥90	1	Reference		Stage 1: ≥90	1	Reference	
Stage 2: 60–89	1.09	0.54–2.23	0.8093	Stage 2: 60–89	1.67	0.86–3.23	0.1270
Stage 3a: 45–59	1.49	0.70–3.18	0.2978	Stage 3a: 45–59	2.14	1.04–4.39	0.0379
Stage 3b: 30–44	3.42	1.64–7.12	0.0010	Stage 3b: 30–44	4.43	2.24–8.76	<0.0001
Stage 4: 15–29	3.60	1.72–7.53	0.0007	Stage 4: 15–29	5.14	2.57–10.28	<0.0001
Stage 5: <15	5.39	2.26–12.88	0.0001	Stage 5: <15	8.00	3.62–17.68	<0.0001

Adjusted model 4			<0.0001	Adjusted model 4			<0.0001
Stage 1: ≥90	1	Reference		Stage 1: ≥90	1	Reference	
Stage 2: 60–89	1.25	0.56–2.81	0.5848	Stage 2: 60–89	1.90	0.91–3.97	0.0874
Stage 3a: 45–59	1.44	0.60–3.46	0.4159	Stage 3a: 45–59	2.12	0.94–4.75	0.0688
Stage 3b: 30–44	3.22	1.40–7.43	0.0060	Stage 3b: 30–44	4.03	1.88–8.63	0.0003
Stage 4: 15–29	3.22	1.43–7.71	0.0051	Stage 4: 15–29	4.77	2.19–10.37	0.0001
Stage 5: <15	6.71	2.51–17.91	0.0001	Stage 5: <15	9.82	4.05–23.80	<0.0001

CKD: chronic kidney disease; HR: hazard ratio; CI: confidence interval; MDRD: modification of diet in renal disease; CKD-EPI: chronic kidney disease epidemiology collaboration.

Model 1: adjusted for age and sex.

Model 2: adjusted for age, sex, and duration of diabetes.

Model 3: adjusted for age, sex, duration of diabetes, and baseline HbA1c level.

Model 4: adjusted for age, sex, duration of diabetes, baseline HbA1c level, and medication for hypertension.
